# Encapsulation of Cinnamic Acid on Plant-Based Proteins: Evaluation by HPLC, DSC and FTIR-ATR

**DOI:** 10.3390/plants10102158

**Published:** 2021-10-11

**Authors:** Mirela Kopjar, Ivana Buljeta, Ivana Jelić, Vanja Kelemen, Josip Šimunović, Anita Pichler

**Affiliations:** 1 Faculty of Food Technology Osijek, Josip Juraj Strossmayer University of Osijek, Franje Kuhača 18, 31 000 Osijek, Croatia; ivana.buljeta@ptfos.hr (I.B.); ijelic@ptfos.hr (I.J.); anita.pichler@ptfos.hr (A.P.); 2 Teaching Institute of Public Health Osijek-Baranja County, Franje Krežme 1, 31 000 Osijek, Croatia; vanya.kelemen@gmail.com; 3 Department of Food, Bioprocessing and Nutrition Sciences, North Carolina State University, Raleigh, NC 27695, USA; simun@ncsu.edu

**Keywords:** cinnamic acid, plant-based protein matrices, HPLC, DSC, FTIR-ATR

## Abstract

Plant-based protein matrices can be used for the formulation of delivery systems of cinnamic acid. Pumpkin, pea and almond protein matrices were used for the formulation of dried complexes. The matrices were used in varying amounts (1%, 2%, 5% and 10%) whilst the amount of cinnamic acid was maintained constant. The obtained complexes were analyzed by HPLC, DSC and FTIR-ATR. The highest amounts of cinnamic acid were determined on complexes prepared by the lowest amounts of protein matrices, regardless of their type. The highest affinity for cinnamic acid adsorption was determined for the pumpkin protein matrix. DSC analysis revealed that adsorption of cinnamic acid caused an increase in the thermal stability of the almond protein matrix, while the other two matrices had the opposite behavior. The complexation of protein matrices and cinnamic acid was proven by recording the IR spectra. The obtained complexes could have potential applications in food products to achieve enrichment with cinnamic acid as well as proteins.

## 1. Introduction

Interactions between phenolics and proteins can occur during food processing but also after the intake of foods. Various interactions cause the formation of protein–phenolic complexes, which can affect the absorption rate of these compounds but also change other properties, depending on the nature of bonding [1]. In regard to proteins, changes in their physico-chemical properties, including nutritional, technological and biological values, can occur since complexes can change protein solubility, digestability and thermal stability [2,3,4]. On the other hand, proteins can reduce the potential health benefits of phenolics by masking their antioxidant potential [4,5]. The positive aspect of these interactions would be the formulation of novel food ingredients/complexes with health benefits. Nowadays, consumer demands are directed towards healthy and naturally functional food products which can provide both nutritional and health-related benefits. Another emerging trend is convenience. Consumers’ demand simplified meal preparation and consumption as well as healthy snacking options in and outside of their homes [4,6,7,8]. With this objective, different food ingredients/complexes were prepared based on different types of proteins and phenolics. Over the last decade, the utilization of plant-based protein matrices for the encapsulation of different phenolic compounds has become quite a popular tool for ensuring their preservation and stability. Plant sources used for the isolation of proteins are soybeans, sunflowers, legume seeds, corn kernels, wheat, quinoa, peas, rice, pumpkin seeds, hemp and peanuts. Their low price and high availability make them desirable additives in the food industry. Additionally, protein isolates can be used in the formulation of foods to improve their nutritional value, but they can also possess emulsifying and gel formation properties [6,8,9,10,11,12,13,14,15,16,17,18].

Cinnamic acid is a natural phenolic acid that is a major component found in cinnamon, as well as in other fruit and vegetables. Through numerous studies, it was proven that this phenolic acid has health benefits, such as antioxidant potential, antimicrobial, anticancer, neuroprotective, anti-inflammatory and anti-diabetic properties [19,20,21,22].

A review of different analyses that can be used for the evaluation of interactions between proteins and phenolics was given by Czubinski and Dwiecki [1]. These analyses include spectroscopic measurements, microscopic, thermodynamic, electrophoretic, chromatographic and bioinformatic analyses. For the evaluation of protein/cinnamic acid complexes, we applied high-performance liquid chromatography (HPLC), differential scanning calorimetry (DSC) and Fourier-transform infrared spectroscopy-attenuated total reflectance (FTIR-ATR). Protein/cinnamic acid complexes were prepared by complexation of different protein matrices (pea, almond and pumpkin) in varying amounts (1%, 2%, 5% and 10%) with cinnamic acid.

## 2. Results and Discussion

Protein complexes were formulated with the complexation of different protein matrices as carriers of cinnamic acid. Varying amounts of carriers and a constant amount of cinnamic acid were used for complexation in order to evaluate their influence on the adsorption of the targeted phenolic acid. Formulated protein complexes were characterized by HPLC, DSC and FTIR analyses.

### 2.1. HPLC Analysis of Protein/Cinnamic Acid Complexes

Results for the amounts of cinnamic acid bound onto protein matrices are presented in Table 1. For the pea protein matrix and almond protein matrix, it was observed that with an increase of carrier, a decrease in adsorption of cinnamic acid occurred; whereas, the almond protein matrix showed slightly different behavior. For this carrier, it was observed that there was no difference between its applications of 1% and 2% for complexation; conversely, with the further increase in its amount, a decrease in adsorption of cinnamic acid occurred. Comparison of the results of complexes prepared with 1% of the carrier indicated that the pumpkin protein complex had the highest amount of cinnamic acid (41.15 mg/g). With the increase of carrier to 2%, there was no difference between pumpkin and almond protein complexes (amount of cinnamic acid was around 34.5 mg/g), while the pea protein complexes formulated with 5% and 10% had higher cinnamic acid amounts (33 mg/g and 30.7 mg/g, respectively) than other complexes. Irrespective of the amount of carriers, the almond protein matrix had the lowest affinity for cinnamic acid.

The characteristics of proteins and phenolics both have an impact on the adsorption of phenolics onto proteins [23]. It was determined that the number of hydroxyl groups and their position had an impact on the reactivity of flavonoids and their binding onto proteins [24]. By comparing the reactivity and strength of the binding of selected phenolic compounds onto soy protein, it was determined that those parameters followed the following order: gallic acid > chlorogenic acid = quercetin > myricetin > caffeic acid > kaempferol > apigenin > flavone. The last two compounds listed had a significantly lower affinity towards soy protein than other phenolics [24]. The capability of the binding of selected phenolic compounds onto albumin and globulin was also evaluated. The results for albumin showed that chlorogenic acid and gallic acid had the highest binding capacity, followed by catechin and quercetin, whereas apigenin and ferulic acid had the lowest binding capacity. A slight difference was observed for globulin. Chlorogenic acid had the highest binding capacity, followed by catechin and gallic acid, then quercetin, and the compounds with the lowest binding capacity were apigenin and ferulic acid. Ferulic acid and apigenin had a significantly lower affinity for binding with the two mentioned protein fractions [23]. However, when the authors investigated the binding of phenolics from extracts of green tea and green coffee on the same protein fractions, a different trend was observed. They observed a higher binding capacity for chlorogenic acid and catechin from extracts to the mentioned protein fractions in comparison to pure compounds. The extracts contained other phenolic compounds that affected the binding of the two mentioned phenolics onto proteins [23], likely due to competition for the same binding sites on proteins.

The encapsulation of phenolics onto proteins, i.e., their interactions, depends on the chemical characteristics of proteins and phenolics but also on conditions in the medium during complexation. Very often, protein matrices contain other organic molecules, such as polysaccharides, so protein content is a very important factor. Additionally, those other molecules can have an impact on encapsulation efficiency [1,4,25]. In our case, the pea protein matrix contained 85% of proteins while the other two matrices contained 50%, and a difference between them was observed. There were other studies emphasizing the importance of protein content on the encapsulation of phenolics. Different protein matrices were used for the encapsulation of phenolics from cranberries. The protein content of applied matrices ranged from approximately 50% (defatted soy flour and medium roast peanut flour) to over 70% (hemp protein isolate, soy protein isolate and pea protein isolate). The highest adsorption capacity for cranberry phenolics was by defatted soy flour, medium roasted peanut flour and hemp protein isolate; therefore, it was not possible to determine a linear correlation between protein content and the adsorption capacity of phenolics [6]. A similar trend was also observed for the adsorption of anthocyanins from blueberry juice on defatted soy flour (47% of proteins), white whole-wheat flour (13% of proteins), brown rice flour (8.6% of proteins) and corn flour (5.3% of proteins) [10]. It was proven that both covalent and/or non-covalent interactions between phenolics and proteins can occur [4,26,27]. Non-covalent binding of phenolics to proteins includes interactions that occur through hydrophobic association, hydrogen bonds, electrostatic attraction and van der Waals forces. The most important non-covalent driving forces for the phenolic–protein complexation are hydrophobic interaction and hydrogen bonds [28].

### 2.2. DSC Analysis of Protein/Cinnamic Acid Complexes

The adsorption of cinnamic acid caused changes to the denaturation temperature of the formulated complexes in comparison to protein matrices. When comparing protein matrices, the highest denaturation temperature was recorded for the pea protein matrix (88.6 °C), followed by pumpkin (87.44 °C) and almond protein matrices (85.24 °C) (Table 2). In contrast to pea and pumpkin protein complexes, adsorption of cinnamic acid had a different effect on almond protein matrices. Pea and pumpkin complexes had lower denaturation temperatures than the corresponding protein matrices. Adsorption of cinnamic acid onto the pea protein matrix caused a greater decrease of the denaturation temperature (85.15–86.88 °C) than onto the pumpkin protein matrix (85.11–86.69 °C). Contrary to these complexes, almond protein complexes had a higher denaturation temperature (86.38–88.18 °C) than the corresponding protein matrix. The results of the impact of interactions between phenolics and proteins on denaturation temperature can be used as a tool for predicting the thermal stability of protein complexes [24]. The greater the decrease of denaturation temperatures of formulated complexes in comparison to proteins means that proteins were less stable in combination with phenolics [29]. Our results are in agreement with other studies that investigated the influence of phenolic compounds on the denaturation temperature of proteins, which proved that the thermal stability of complexes depended on both the type of proteins and the type of phenolics. β-lactoglobulin/green tea polyphenols nanocomplex had a lower denaturation temperature than β-lactoglobulin [30]. The same influence of green tea polyphenols was observed on the denaturation temperature of egg albumen [29]. On the other hand, there are also data reporting improvements to the thermal stability of protein–phenolic complexes. An increase in the denaturation temperatures of complexes of soy protein with phenolic acids, quercetin and myricetin was observed, but there was no change of denaturation temperature when soy protein was complexed with flavone, apigenin and kaempferol [24]. Prigent and co-workers [31,32] also reported an increase in denaturation temperatures of α-lactalbumin, lysozyme and bovine serum albumin upon the binding of chlorogenic acid. It can be concluded that the thermal stability of proteins upon binding of phenolics depended on both the type of protein as well as the type of phenolic. Consequently, we can conclude that almond protein complexes were more thermally stable than the corresponding protein matrix, while the opposite effect was observed for pea and pumpkin protein complexes.

### 2.3. FTIR-ATR Analysis of Protein/Cinnamic Acid Complexes

**Figure 1:**
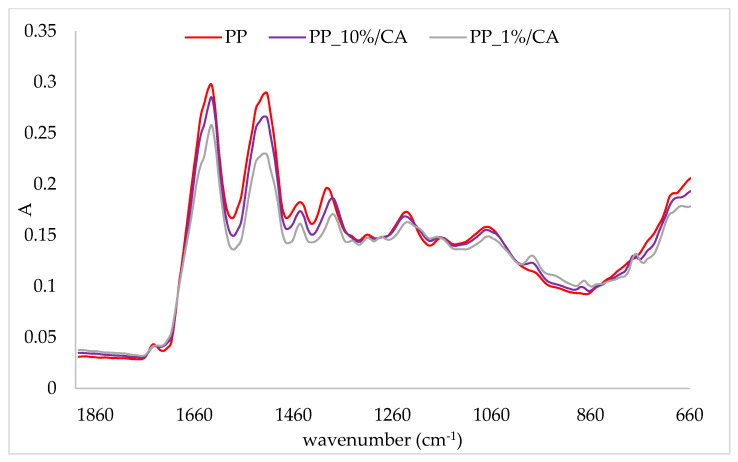
IR spectra of pea protein (PP) and obtained complexes with cinnamic acid (PP_10%CA—complex prepared with 10% of protein matrix; PP_1%CA—complex prepared with 1% of protein matrix).

**Figure 2:**
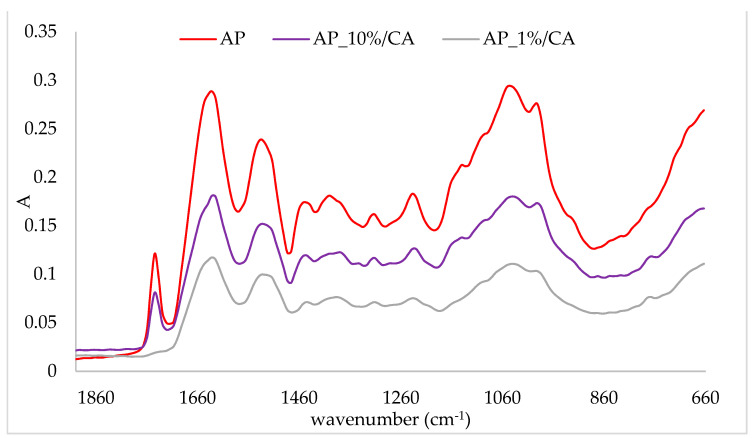
IR spectra of almond protein (AP) and obtained complexes with cinnamic acid (AP_10%CA—complex prepared with 10% of protein matrix; AP_1%CA—complex prepared with 1% of protein matrix).

**Figure 3:**
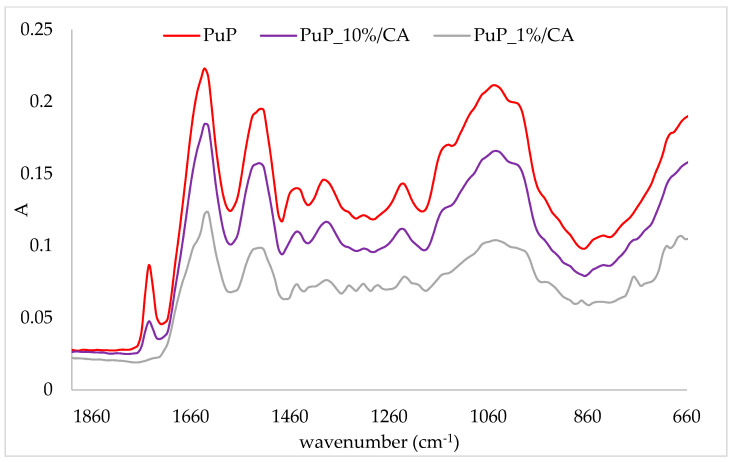
IR spectra of pumpkin protein (PuP) and obtained complexes with cinnamic acid (PuP_10%CA—complex prepared with 10% of protein matrix; PuP_1%CA—complex prepared with 1% of protein matrix).

**Figure 4:**
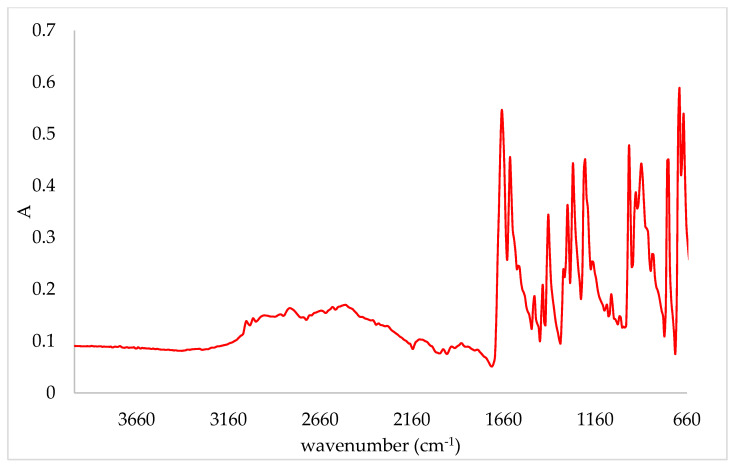
IR spectra of cinnamic acid.

In order to prove the binding of cinnamic acid onto protein matrices, a comparison of the IR spectra of protein complexes and protein matrices was conducted. The obtained IR spectra are presented in Figure 1, Figure 2, Figure 3 and Figure 4. In the figures, the IR spectra of the protein matrix and complexes prepared with the lowest (1%) and the highest (10%) amounts of the corresponding protein matrix are presented for easier comparison. The band intensity of the IR spectra of protein matrices was higher than those of complexes. Additionally, some other changes were recorded on complexes in comparison to protein matrices. Comparison of the IR spectra of complexes for all protein matrices indicated that the lowest band intensity of the IR spectra had complexes prepared with 1% of the protein matrix and the highest with 10% (IR spectra of other two complexes are between those two). Correlation of those results with the amount of cinnamic acid can be observed, i.e., complexes with the highest amount of cinnamic acid had the lowest band intensity, and through the decrease of the amount of cinnamic acid, an increase in band intensity occurred. Additionally, some of the other changes were more pronounced on those complexes which contained the highest amounts of cinnamic acid.

Changes in the IR spectra of pea protein complexes in comparison to the pea protein matrix were observed at several wavenumbers (Figure 1). The band at 1743 cm^−1^, which was assigned to C=O, shifted to 1740 cm^−1^. Pea protein powder had a band at 1395 cm^−1^, which was assigned to symmetric CH_3_ bending of the methyl groups of proteins [33]. Adsorption of cinnamic acid onto the protein caused its shift to 1380 cm^−1^. At complexes with 1% of protein, a band at 1342 cm^−1^ appeared, which was assigned to CH_2_ wagging [33]. On complexes, a shoulder at 1200 cm^−1^ next to the band at 1232 cm^−1^ appeared. Additionally, the band at 1160 cm^−1^, assigned to the stretching vibrations of hydrogen bond of C-OH groups [33], shifted on complexes at 1165 cm^−1^ and 1170 cm^−1^ for complexes with 10% and 1% of protein, respectively. Additional changes on the pea protein matrix due to the adsorption of cinnamic acid were also observed. Three bands of low intensity appeared at 980 cm^−1^, 874 cm^−1^ and 770 cm^−1^, which all originated from cinnamic acid as visible from the comparison to its own IR spectra (Figure 4).

The adsorption of cinnamic acid onto the almond protein matrix also caused changes in its IR spectra (Figure 2). The band at 1745 cm^−1^, which was assigned to C=O, disappeared on the complex prepared with 1% of protein. Additionally, two other bands which were assigned to Amid I and Amid II regions shifted. The band at 1635 cm^−1^ shifted to 1630 cm^−1^ and the band at 1535 cm^−1^ to 1530 cm^−1^. Amid bands, which are characteristic for proteins, are Amid I (C-O stretching) and Amid II (N-H bending and C-H stretching), which were assigned to regions 1700–1600 cm^−1^ and 1600–1500 cm^−1^, respectively [34,35,36]. Similar to pea protein complexes, a band at 770 cm^−1^ was formed upon adsorption of cinnamic acid.

A comparison of the IR spectra of pumpkin complexes and the corresponding protein matrix is presented in Figure 3. Similar to almond samples, a band at 1745 cm^−1^ disappeared on the complex prepared with 1% of protein. Another change was observed on a band at 1630 cm^−1^; after the adsorption of cinnamic acid, a shoulder appeared at 1655 cm^−1^. Additional bands were formed, but they were only visible on the complex with 1% of protein. Those bands were at 1342 cm^−1^, 1315 cm^−1^, 1285 cm^−1^, 874 cm^−1^ and 770 cm^−1^.

Plant proteins differ in their structure [37,38]. Depending on the structure of plant proteins, as well as the structure of phenolics, different structural changes on the IR spectra can be observed [39,40], as was the case with the complexes prepared in this study.

## 3. Materials and Methods

### 3.1. Materials

Cinnamic acid was purchased from Fisher Scientific (Loughborough, UK) and the proteins were a donation from Blesterfeld (Germany). Orthophosphoric acid (HPLC grade) was from Fisher Scientific (Loughborough, UK). Methanol (HPLC grade) was from Avantor Performance Materials (Gliwice, Poland).

### 3.2. Formulation of Protein/Cinnamic Acid Complexes

The complexes were prepared by complexation of different types of protein matrices in the amount of 1%, 2%, 5% or 10% with 20 mL of cinnamic acid (2 mM). Sources of proteins were pea (approximately 85% of proteins), almond and pumpkin (approximately 50% of proteins) matrices. For the preparation of protein/cinnamic acid complexes, a defined amount of each protein matrix and solution of cinnamic acid was stirred for 15 min on a magnetic stirrer (600 rpm) at room temperature. The prepared mixture was then centrifuged for 15 min at 4000 rpm, and the wet-solid phase was separated and dried. The obtained dry powder presented the protein/cinnamic acid complex.

### 3.3. Reverse-Phase High Performance Liquid Chromatography (RP-HPLC)

Prior to the HPLC analyses, the complexes were extracted. The formulated protein/cinnamic acid complexes (0.15 g) were extracted with 10 mL of acidified methanol (HCl:methanol ratio was 1:99) for 24 h at room temperature, and the obtained extracts were then filtered and used for the evaluation of cinnamic acid amounts. The amount of cinnamic acid was analyzed with RP-HPLC system 1260 Infinity II (Agilent technology, Santa Clara, CA, USA). The system consisted of a quaternary pump, diode array detector (DAD) and Poroshell 120 EC-C 18 column (4.6 × 100 mm, 2.7 µm). Orthophosphoric acid (0.1%) as mobile phase A and methanol (100%) as mobile phase B were used. For separation, the following gradient was used: 0 min 5% B, 3 min 30% B, 15 min 35% B, 22 min 37% B, 30 min 41% B, 32 min 45% B, 40 min 49% B, 45 min 80% B, 48 min 80% B, 50 min 5% B and 53 min 5% B. The injection volume was 10 µL, and the flow rate was set to 1 mL/min. A calibration curve for cinnamic acid was generated for the range from 25 to 300 mg/L (r^2^ = 0.9983; LOD = 3.76 mg/L; LOQ = 11.38 mg/L; RSD = 0.84%; recovery 103,52%). UV/Vis spectra were recorded in a range between 190 and 600 nm. Measurements were conducted in duplicate.

### 3.4. Analysis by Differential Scanning Calorimetry (DSC)

DSC analysis was carried out on a differential scanning calorimeter, Mettler Toledo 822. (Mettler Toledo, Switzerland). Each sample (7 ± 0.5 mg) was weighted in a 40 µL aluminum pan with a cover. The aluminum pans were transferred into the oven of the DSC instrument, and recordings were carried out in a temperature range from 25 °C to 140 °C. At 25 °C, samples were tempered for 4 min. Afterwards, the temperature was increased to 140 °C at the rate of 5 °C per minute. After reaching the final temperature (140 °C), samples were tempered for 4 min. Measurements were conducted in duplicate.

### 3.5. Recording of IR Spectra by Fourier-Transform Infrared Spectroscopy-Attenuated Total Reflectance (FTIR-ATR)

FTIR-ATR was used for the screening of the IR spectra of the protein matrices and protein matrices loaded with cinnamic acid. The IR spectra analyses were carried out from 4000 to 600 cm^−1^ on a Cary 630 FTIR spectrometer (Agilent technology, Santa Clara, CA, USA).

### 3.6. Statistical Analysis

The obtained results were analyzed using the software program STATISTICA 13.1 (StatSoft Inc, Tulsa, OK, USA), using the variance analysis (ANOVA) and Fisher’s least significant difference (LSD) with significance defined at *p* < 0.05. All results were expressed as mean value ± standard deviation.

## 4. Conclusions

Plant-based protein matrices can be used for the encapsulation of cinnamic acid. Complexation of different protein matrices and cinnamic acid was proven by HPLC, DSC and FTIR-ATR analyses. Based on our results, it can be concluded that the highest affinity for cinnamic acid adsorption was determined for the pumpkin protein matrix. Additionally, with the increase in the amount of the protein matrix during complexation, a decrease in the adsorption of cinnamic acid was observed. The obtained complexes could have potential applications in food products to achieve enrichment with cinnamic acid as well as proteins.

## Figures and Tables

**Table 1:** Amounts of cinnamic acid (mg/g) on protein complexes.

Amount of Protein Matrix	Protein Matrix		
	Pea	Almond	Pumpkin
1%	35.82 ± 0.05 ^a^	30.37 ± 0.47 ^a^	41.15 ± 0.11 ^a^
2%	34.95 ± 0.24 ^a^	30.49 ± 0.13 ^a^	34.13 ± 0.67 ^b^
5%	32.99 ± 0.16 ^b^	23.63 ± 0.17 ^b^	29.05 ± 0.02 ^c^
10%	30.69 ± 0.63 ^c^	20.67 ± 0.01 ^c^	25.58 ± 0.02 ^d^

Values in the same column marked with different letters were significantly different.

**Table 2:** Denaturation temperatures of protein matrices and protein complexes.

Amount of Protein Matrix	Protein Matrix		
	Pea	Almond	Pumpkin
100%	88.60 ± 0.47 ^a^	85.24 ± 0.07 ^d^	87.44 ± 0.27 ^a^
1%	85.15 ± 0.39 ^c^	88.18 ± 0.08 ^a^	85.11 ± 0.25 ^c^
2%	85.26 ± 0.44 ^c^	87.19 ± 0.20 ^b^	86.13 ± 0.47 ^b^
5%	85.66 ± 0.38 ^c^	86.73 ± 0.12 ^b,c^	86.30 ± 0.34 ^b^
10%	86.88 ± 0.33 ^b^	86.38 ± 0.36 ^c^	86.69 ± 0.39 ^b^

Values in the same column marked with different letters were significantly different.

## Data Availability

Not applicable.
